# Anti-inflammatory effect of Ganluyin, a Chinese classic prescription, in chronic pharyngitis rat model

**DOI:** 10.1186/s12906-020-03057-5

**Published:** 2020-08-28

**Authors:** Ye-Hui Chen, Rong Luo, Shan-Shan Lei, Bing Li, Fu-Chen Zhou, Hui-Ying Wang, Xue Chen, Xinglishang He, Yu-Zhi Wang, Liang-Hui Zhan, Ting-Ting Lu, Jie Su, Qiao-Xian Yu, Bo Li, Gui-Yuan Lv, Su-Hong Chen

**Affiliations:** 1 Zhejiang University of Technology, Hangzhou Zhejiang, 310014 People’s Republic of China; 2 Zhejiang Chinese Medical University, Hangzhou Zhejiang, 310053 People’s Republic of China; 3 Zhejiang Senyu Co., Ltd, Yiwu, Zhejiang, 322099 People’s Republic of China

**Keywords:** Ganluyin, Chronic pharyngitis, Anti-inflammation, Pro-inflammatory cytokines

## Abstract

**Background:**

Ganluyin (GLY) is a famous classical prescription with a long history of use as a treatment for inflammatory conditions such as chronic pharyngitis (CP) in many parts of China. However, it has not been developed as a modern pharmaceutic and its anti-inflammatory mechanisms remain unclear. The aim of this study was to assess the anti-inflammatory efficacy of GLY and potential mechanisms in a rat model of CP.

**Methods:**

The chemical profile of GLY was analyzed by HPLC-UV. We used a mouse model of ear edema and a rat model of paw edema. Specifically, xylene was used to induce edema on the surface of one ear in mice, and carrageenan was injected subcutaneously into the right hind paws of rats to induce paw edema. The paw thickness, ear weight, and ear perfusion were measured and recorded. The CP model in rats was induced by irritating the throat with 5% ammonia and was used to evaluate the therapeutic efficacy of GLY. Levels of interleukin-6 (IL-6), interleukin-1β (IL-1β), tumor necrosis factor (TNF-α), and prostaglandin E2 (PGE2) were measured by ELISA in serum, and protein expression of cyclooxygenase-2 (COX-2) and nuclear factor kappa-B p65 (NF-κB p65) in the throat were detected by immunohistochemistry and Western blot to evaluate the anti-inflammatory mechanism of GLY. Hematological assays were also conducted.

**Results:**

There were four flavonoids identified in GLY: naringin, neohesperidin, baicalin, and wogonoside. The oral administration of GLY showed a significant inhibitory effect on xylene-induced ear swelling and ear blood flow in mice and significantly ameliorated rat right hind paw edema at doses of 6.2 and 12.4 g/kg. Mechanistic studies found that the anti-inflammatory activity of GLY was related to the inhibition of pro-inflammatory cytokines such as IL-1β, IL-6, TNF-α, and PGE2 and that GLY reduced the expression of COX-2 and NF-κB p65 proteins in the throat, attenuated throat injury, and reduced inflammatory exudates. Hematological analysis showed that treatment with GLY prevented increases in white blood cell (WBC), neutrophil (NEUT), lymphocyte (LYMPH) and monocyte (MONO) levels.

**Conclusions:**

These studies indicated that GLY has beneficial anti-inflammatory effects on CP and that it acts through reducing pro-inflammatory factors such as IL-1β, IL-6, TNF-α, and PGE2, as well as decreasing WBC, NEUT, LYMPH and MONO levels and decreasing the expression of COX-2 and NF-κB p65 proteins. These findings may lay the groundwork for further studies of GLY as a suitable candidate for the treatment of inflammatory diseases such as CP.

## Background

Chronic pharyngitis (CP), a global disease, is a condition describing chronic inflammation of the pharynx and submucosal lymphoid tissues and is a disease that is commonly seen in otorhinolaryngology clinics across the world [1]. Pharyngitis in high-income countries accounts for 22.6% ~ 26.1% of outpatient clinic visits [2]. Indeed, the number of outpatient visits with a primary diagnosis of CP has been estimated to be as high as 7 million to 11 million visits per year in the United States [3]. In China, CP patients make up as many as one-third of all visits to otorhinolaryngology clinics [4]. Environmental pollution, microbial pollution, and smoking can exacerbate the occurrence of chronic pharyngitis [5]. For example, in a study of 2064 urban residents from cities with severe air pollution, the prevalence of CP has been recorded as high as 81.9%, posing serious implications for human physical and mental health as well as life satisfaction [6, 7]. More significantly, persistent inflammation may render the nasopharyngeal mucosa susceptible to carcinogenesis [8]. The exacerbation of inflammation is important for continued pathology, so breaking the chain of persistent inflammation may enable effective prophylaxis and treatment of CP [9].

Models of CP are typically established using irritants such as ammonia, sand, and capsaicin, which are used to simulate environmental pollution or unhealthy human lifestyles [10]. The current clinical treatment of CP mainly revolves around the use of glucocorticoids, antibiotics, and nonsteroidal anti-inflammatory drugs (NSAIDs) [11, 12]. However, extended treatment of CP with drugs such as aspirin or dexamethasone could cause gastric mucosal, liver, or kidney damage as well as other side effects that are not tolerable. According to prior studies, many traditional Chinese medicines (TCMs) have a beneficial role in CP, with the potential to reduce excessive inflammatory progression and restore throat tissue homeostasis [13, 14].

Ganluyin (GLY) is a classic and famous Chinese medicine prescription composed of a combination of ten TCMs, including the root of *Scutellaria Baicalensis*, *Rehmannia Glutinosa*, *Radix Rehmanniae Praeparata*, *Asparagus Cochinchinensis*, *Ophiopogon japonicus* and *Glycyrrhiza Uralensis*, the stem of *Dendrobii Caulis*, the aboveground of *Artemisia Capillaris*, the leaf of *Eriobotrya japonica*, the fruit of *Fructus Aurantii*, and has been widely used to treat sore throats clinically in China [15]. The composition and details of GLY are displayed in Table S1. The components of GLY have been shown to have significant protective effects against inflammation in laboratory animals and human throat diseases (ulcers, sore throat, cough, etc.) [16, 17]. Further, GLY has a broad-spectrum bacteriostatic effect, inhibiting food bacteria and pathogens that typically infect the throat. GLY has been shown to inhibit a variety of viruses through an inhibitory effect on virus infection and proliferation. A previous study showed that GLY restores proper TNF-α levels in human oral cancer cells by acting on nuclear factor kappa-B (NF-κB), protein kinase B (AKT), and extracellular regulated protein kinases (ERK)-dependent pathways [18]. The GLY formula has been widely used to treat sore throat clinically in China, with high cure rates for treatment of CP in clinical studies [19, 20]. In addition, some of the components in GLY such as Dendrobium officinale polysaccharide (DOP) and *Ophiopogon japonicus* have been noted to bolster immunity and have analgesic effects. Jian Liang [21] found that DOP reduces the secretion of nucleotide domain-like receptor protein 3 (NLRP3) inflammatory bodies in vivo and in vitro by blocking the β-arrestin1 signaling pathway, leading to a therapeutic effect on ulcerative colitis (UC). Network pharmacology studies have suggested that baicalin inhibits the production of inflammatory factors, inhibits the binding of inflammatory factors to their corresponding receptors, and blocks the initiation of inflammatory responses through mitogen-activated protein kinase 14 (MAPK14), epidermal growth factor receptor (EGFR), tumor necrosis factor receptor super family 1A (TNFRSF1A), E-Selectin (SELE) and other targets [22]. The combination of Radix Scutellariae and *Ophiopogon japonicus* has been shown to treat recurrent oral ulcers by regulating the balance of T lymphocyte subsets [23]. Combined, these prior insights suggest that the classic prescription of GLY may have the potential as the basis for new therapeutics to treat inflammatory conditions in the throat.

Although this formula has a longstanding clinical history of use for CP [15], its mechanism of action is still unclear. Thus, we studied the potential anti-inflammatory activity of GLY to clarify the therapeutic mechanism of CP and to support the potential clinical applications of GLY. In this research, GLY was administered in an ammonia-induced CP rat model. Serum levels of the pro-inflammatory cytokines IL-1β, IL-6, TNF-α, and PGE2 were measured by ELISA, and expression of COX-2 and NF-κB p65 proteins in the throat were detected by Western blot and immunohistochemistry to assess the anti-inflammatory mechanism of GLY. Simultaneously, the anti-inflammatory effects of GLY were evaluated through an ear and foot edema assay in two animal models of acute inflammation.

## Methods

### Animals

Male Institute of Cancer Research (ICR) mice weighing 20 ~ 23 g and male Sprague Dawley (SD) rats weighing 180 ~ 200 g were purchased from Zhejiang Academy of Medical Sciences (SCXK2019–0002, Zhejiang, China), and raised in the animal room of Zhejiang University of Technology. All experiments about animals in this study are abided by moral and legal requirements. All course were abided by regulations on the Administration of Laboratory Animals issued by the Ministry of Science and Technology of China, and the operations were given a licence the Medical Ethics Committee of Zhejiang University of Technology (SYXK (Zhe) 2017–0001).

The animal specimens were euthanized after completing the experiment, anesthetized rats by intraperitoneal inject 2% pentobarbital sodium, and the injection dosages was 0.3 mL/100 g, which was proved to be applicable through literature review and previous experimental experience.

### Chemicals and reagents

The ELISA kits of IL-6, IL-1β, TNF-α, and PGE2 were purchased from Shanghai Enzyme-linked Biotechnology Co., Ltd. (Shanghai, China). Dexamethasone was purchased from Shanghai Shangyao Xinyi Pharmaceutical Factory Co., Ltd. (Shanghai, China). Carrageenan and aspirin were purchased from Macklin Reagent Co., Ltd. (Shanghai, China). Xylene was obtained from Hangzhou Shuanglin Chemical Co., Ltd. (Hangzhou, China). Ammonia was purchased from Hangzhou Longshan Fine Chemical Co., Ltd. (Hangzhou, China). Citrate buffer solution and DAB were obtained from Beyotime Biotechnology Reagent Co., Ltd. (Zhejiang, China). BSA and anti-rabbit IgG were obtained from Boster Biological Technology Co., Ltd. (Zhejiang, China). COX-2, NF-κB p65, and GAPDH antibodies were purchased from Proteintech Biotechnology Co., Ltd. (Zhejiang, China).

### Preparation of GLY extract and HPLC-UV analysis

The 10 herbs of GLY were purchased from Zhejiang Inte Pharmaceutical Co., Ltd. All the herbs were soaked for 12 h in pure water, which is 10 times their weight and then decocted for 1 h. After obtaining the filtrate, the remaining residue is boiled and filtered again. Combining the two filtrates and concentrate (60 °C) the filtrate into thick paste with the concentration of 0.37 g /mL GLY solution (crude herb).

The quality of GLY was determined by HPLC equipped with Ultimate LP-C18 column (4.6 mm × 250 mm, 5 μm). Detection wavelength was set at 280 nm. The mobile phase was consisted of methyl alcohol (MeOH) (A) and 0.2% phosphoric acid solution (B) and the gradient elution procedure was as follow: 0 ~ 5 min, 75% B; 5 ~ 10 min, 75 ~ 70% B; 10 ~ 25 min, 70 ~ 60% B; 25 ~ 45 min, 60 ~ 45% B; 45 ~ 60 min, 45 ~ 30% B; 60 ~ 75 min, 30% ~ 75% B; flow rate: 1 mL/min; injection volume: 10 μL. Naringin, neohesperidin, baicalin, and wogonoside were set as the quality control to make the chemical components of GLY of different batches nearly the same.

### Feet swelling assay

Sixty SD rats were divided into 6 groups (*n* = 10), including the normal group (NG), model group (MG), dexamethasone group (Dex, 1.4 mg/kg), GLY high dose group (GLY-H, 12.4 g/kg), GLY middle dose group (GLY-M, 6.2 g/kg), and GLY low dose group (GLY-L, 3.1 g/kg). Rats were orally administered once a day for one month and 1 h after the last dose. First, 0.1 mL of physiological saline (0.9%, w/v) with newly prepared carrageenan (1%, w/v) was injected into the right hind paw plantar tissues of each rat except the normal group (which receive 0.1 mL saline) to induce inflammation. As a control, the left hind paws were not injected. A vernier caliper was used to measure the thickness of both hind paws of each animal at 1 h before induction and 1 ~ 6 h after induction. Calculating the increase rate that in paw thickness of the right hind paw (foot swelling) was calculated as: the increase rate = (B-A) / A × 100%, where A represents the paw thicknesses before induction and B represents the paw thicknesses at different time points after the induction [24].

### Ear swelling assay

Sixty ICR mice were divided into 6 groups (*n* = 10), including normal group (NG), model group (MG), dexamethasone group (Dex, 2.2 mg/kg), GLY high dose group (GLY-H, 12.4 g/kg), GLY middle dose group (GLY-M, 6.2 g/kg), and GLY low dose group (GLY-L, 3.1 g/kg). Mice were orally administered once a day for one month and 1 h after the last dose. To induce inflammation, both sides of each mouse right ear except normal group was induced by applying 20 μL xylene, and as controls, the left ear of rats were not given any dispose. 0.5 h later, anesthetize mouse using a respiratory anesthesia machine and photograph auricle microcirculation. Then, we cut the ears along the base line of the auricle, and used the ear piercer to punch a small round piece at the same part of left and right ear and weigh them. The definition of edema was the weight difference between the two ears.

### Establishment of CP model in rats and animal treatment

Fifty male rats were randomly divided into a normal group (*n* = 10) and control group (*n* = 40) according to body weight, and 5% ammonia water was sprayed into the throat of the model rat with a throat atomizer (2 times/d, 3 s/time, 8 h apart), the normal group was sprayed with the same amount of distilled water.

After 15 days, the orbital venous plexus of the rats was operated to collect blood samples after fasting for 12 h, which was used for haematological analysis. Then, the control group was randomly divided into 4 groups (*n* = 10), including the model group (MG), aspirin group (50 mg/kg, Aspirin), GLY high dose group (GLY-H, 12.4 g/kg), and GLY low dose group (GLY-L, 6.2 g/kg) based on rat weight and WBC counts. The rats were given the corresponding drug by gavage for 15 days.

All groups were simultaneously administered from the 16th day (1.0 mL/100 g, 15 days consecutively). We randomly anesthetized three rats in each group by a respiratory anesthesia machine every three days and observed the changes in the pharyngeal tissue.

### Macroscopic observation

Before the end of experiment, we anesthetized all rats and observed the changes in the pharyngeal tissue and assigned scores under blinded condition according to Table 1 [25].

**Table 1:** The scores of throat tissue

Score	Appearance
0	Throat tissue is light red, the surface is moist and shiny, no secretions, no congestion, swelling and other pathological phenomena.
1	Bad gloss of throat tissue, a small amount of secretion, a small amount of acute congestion.
2	Tissue color dark red, poor color, secretion, and accompanied by acute, mild swelling and other phenomena.
3	Pharynx tissue is dark red and dark, mucus secretion is greatly increased, acute congestion, swelling is obvious

### Haematological analysis

The orbital venous plexus of the rats was operated to collect blood samples on the 29th day. In order to avoid clotting, blood samples were stored in Ethylene Diamine Tetraacetic Acid (EDTA) coated tubes. Sysmex haematology systems analyzer was used to analysis haematological parameters. The results of complete blood counts (NEUT, WBC, LYMPH and MONO) and differentials were used to assess the therapeutic effect of research parameters.

### Hematoxylin and eosin (H&E) staining

Ear, throat, and lung tissue sections were Hematoxylin and eosin (H&E) staining according to the previously published literatures [26]. The results were assessed by Olympus BX43 light microscope (Olympus, Tokyo, Japan) and the throat pathology of pharyngitis was scored as Table 2 described [27]. The thicknesses of the ear and throat were determined from six points by the Image-Pro Plus software.

**Table 2:** The scores of H&E of the throat

Score	Appearance
0	Normal structure
1	Throat congestion, less inflammation
2	Limited pharyngeal inflammation with spike formation
3	Pharyngeal inflammation is diffuse and subepithelial studs are obvious

### Measurement of the levels of IL-1β, IL-6, TNF-α, and PGE2 in serum

At the end of the experiment, blood was collected by abdominal aorta puncture under general anesthesia of rats. After water bathed for 1 h, the samples were centrifuged for 10 min at 3000 rpm and separated to obtain serum. Then measuring the levels of IL-6, IL-1β, PGE2 and TNF-α in rat serum by ELISA kits according to the instructions.

### Immunohistochemical staining

The expression and localization of NF-κB p65 and COX-2 in throat were determined by immunohistochemical staining according to our previous literature [28]. The tissue sections were incubated with COX-2 (1:200, dilution) or NF-κB p65 (1:200, dilution) primary antibody. All staining was photographed with the microscope, and the protein expression datas in the positive region of the micrograph was analyzed IOD semi-quantitatively by Image Pro Plus software.

### Western blot analysis

The throat tissues were mechanically grinded in a glass homogenizer, the RIPA lysis buffer (Beyotime, Jiangsu, China) and protein phosphatase inhibitor (Beyotime, Jiangsu, China) were added to lysed the samples for 1 h on ice. In order to acquire supernatant, homogenates were centrifuged for 10 min at 12,000 rpm and 4 °C [29]. BCA protein test kit (Beyotime, Jiangsu, China) was used for the protein quantification. Then boiling the soluble extracts with Loading buffer (Beyotime, Jiangsu, China) for 10 min. The equal amount of protein exact of samples were electrophoresed on a 10% SDS-PAGE, then the electrophoretic proteins are transferred onto a PVDF. After blocking, membrane was probed with goat polyclonal antibodies against NF-κB p65 (1:2000; Proteintech, USA), COX-2 (1:1000; Proteintech, USA), GAPDH (1:5000; Proteintech, USA). The second day, rinsing the membrane with tris-buffered saline Tween (TBST) 3 times for 10 min, and incubating with goat anti-rabbit IgG (1:5000; Proteintech, USA), which can conjugate with appropriate secondary antibody HRP. Afterwards, cleaning the membrane again with TBST and treated with a chemiluminescence analysis kits (Beyotime, Jiangsu, China) for visualization. The bands were analyzed (relative to GAPDH expression) by using Gel-pro analyzer [30].

### Statistical analysis

All analyses were performed using the updated version of SPSS software. All data were expressed as the means ± SEM and the differences were subjected to the t-test. Diagrams were prepared applying the Graph Prims.

## Results

### HPLC-UV analysis of GLY

**Fig. 1:**
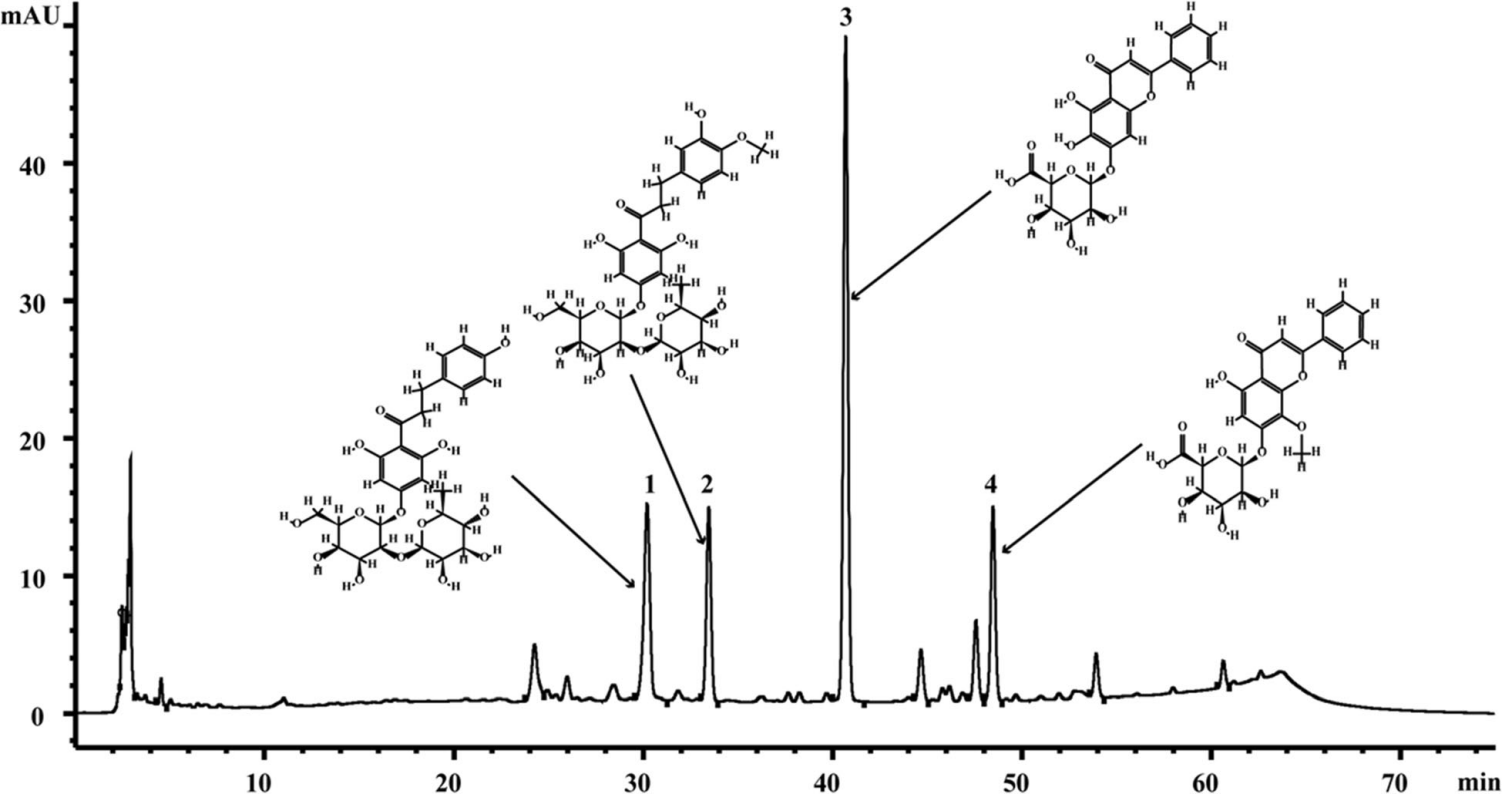
GLY water extract. 1 = naringin, 2 = neohesperidin, 3 = baicalin, 4 = wogonoside with retention time 30.208 min, 33.465 min, 40.697 min, 48.476 min, respectively

HPLC-UV analysis of the GLY water extract (Fig. 1) identified four flavonoids (naringin, neohesperidin, baicalin, and wogonoside) by comparing retention times against verified authentic references. The contents of these flavonoids, naringin, neohesperidin, baicalin, and wogonoside, were 1.63±0.05, 1.30±0.03, 2.47±0.03, and 0.97±0.03 mg/mL, respectively (Table 3).

**Table 3:** Amounts of compounds 1–4 in the GLY

Standard	Amount of Extracts (mg/mL)
1. naringin	1.63 ± 0.05
2. neohesperidin	1.30 ± 0.03
3. baicalin	2.47 ± 0.03
4. wogonoside	0.97 ± 0.03

### Inhibition of paw edema of rats by treatment of GLY

**Fig. 2:**
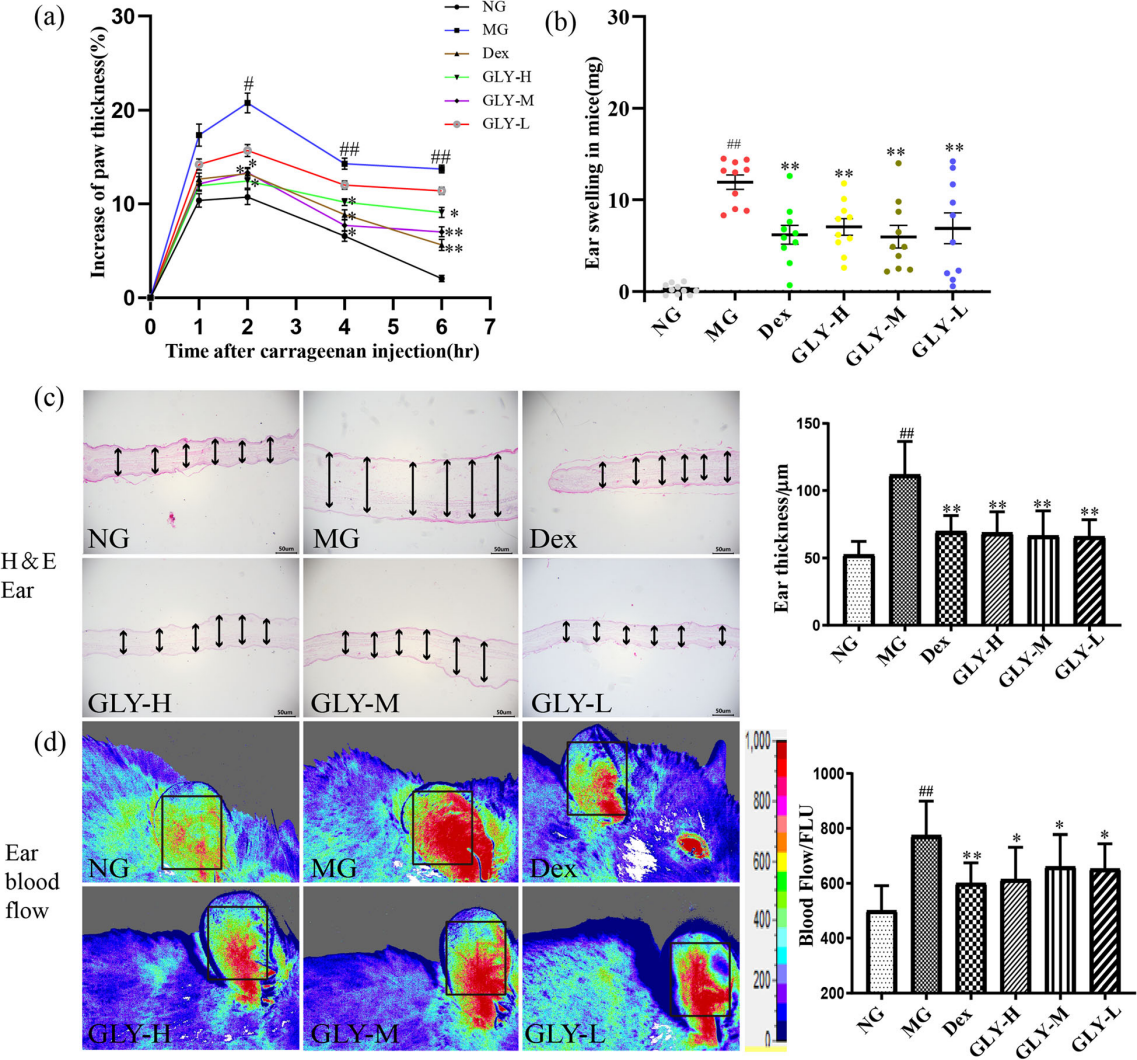
Effect of GLY on anti-inflammation. (**a**) Inhibition of carrageenan-induced paw edema of rats by treatment of GLY. (**b**) Inhibition of xylene-induced ear edema of rats by treatment of GLY. (**c**) Ear was stained with H&E to observe ear thickness and the statistical analysis of them. (**d**) Ear blood flow and the statistical analysis of them. ^#^*p* < 0.05, ^##^*p* < 0.01 compared with normal group; *p < 0.05, **p < 0.01 compared with model group, *n* = 10

In a paw edema model caused by carrageenan injection, GLY can markedly inhibit acute paw edema (Fig. 2a). In this study, the model group had a significant inflammatory reaction after 2 h (*P* <  0.05, 0.01). At the same time point, compared with the model group, the GLY-H and GLY-M groups showed significant improvement in paw edema at 2–6 h (*P* < 0.05, 0.01). This suggests that GLY has significant anti-inflammatory activity in this model of paw edema in rats.

### Inhibition of ear edema of mice by treatment of GLY

Next, we sought to assess the ability of GLY to attenuate edema in the ears of mice, in a model induced by xylene. The activity of GLY in a xylene-induced model of ear edema is shown in Fig. 2. Compared with the model group, the degree of ear swelling induced by xylene was significant reduced in the treated group (*P* < 0.01) (Fig. 2b). We found that the ear of model mice was much thicker, with a 108.3% increase in thickness, relative to normal mice. However, the ear edema was significantly reversed by GLY treatment (*P* < 0.01) (Fig. 2c). As expected, the measured auricular blood flow in the model group increased significantly compared with the normal group (*P* < 0.01). Similarly, oral treatment with GLY showed significant improvement in decreasing the auricle blood flow compared with the model group (*P* < 0.05) (Fig. 2d).

### GLY attenuated the throat apparent and hematology in ammonia-induced CP rats

**Fig. 3:**
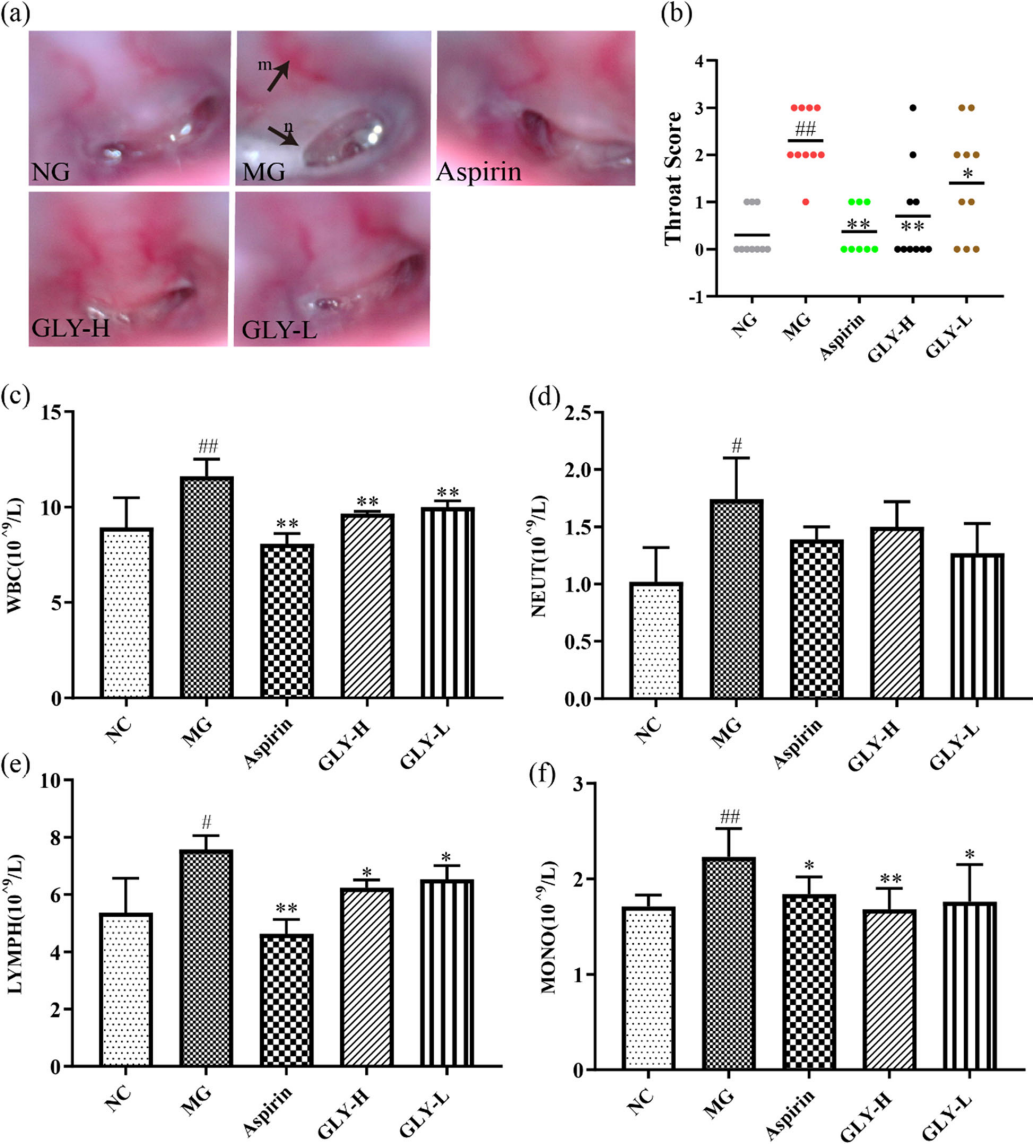
Effects of GLY on the throat apparent and haematology. (**a**, **b**) Appearance of throat and the analysis of them. (**c**) Counts of WBC. (**d**) Counts of NEUT. (**e**) Counts of LYMPH. (**f**) Counts of MONO. ^#^*p* < 0.05, ^##^*p* < 0.01 compared with normal group; *p < 0.05, **p < 0.01 compared with model group, n = 10

Compared with the normal group, there was obvious dilation of capillaries and increased secretion of mucus, with the formation of local ulcers in the ammonia-induced rat model of CP, but this effect was significantly reversed in groups that were administered with GLY-H and GLY-L (Fig. 3a). Compared with the model group, GLY-H and GLY-L also significantly decreased the throat pathology score (*P* < 0.05, 0.01) (Fig. 3b).

Compared with the normal group, the WBC, NEUT, LYMPH, and MONO counts of the model group were significantly enhanced (*P* < 0.05, 0.01) (Fig. 3c-f). Compared with the model group, WBC, LYMPH and MONO counts in the GLY-H and GLY-L groups were significantly improved (*P* < 0.05, 0.01) (Fig. 3c, e and f). Thus, pathological changes were significantly protected against by treatment with GLY-H and GLY-L.

### GLY improved the throat pathology in the ammonia-induced CP rats

**Fig. 4:**
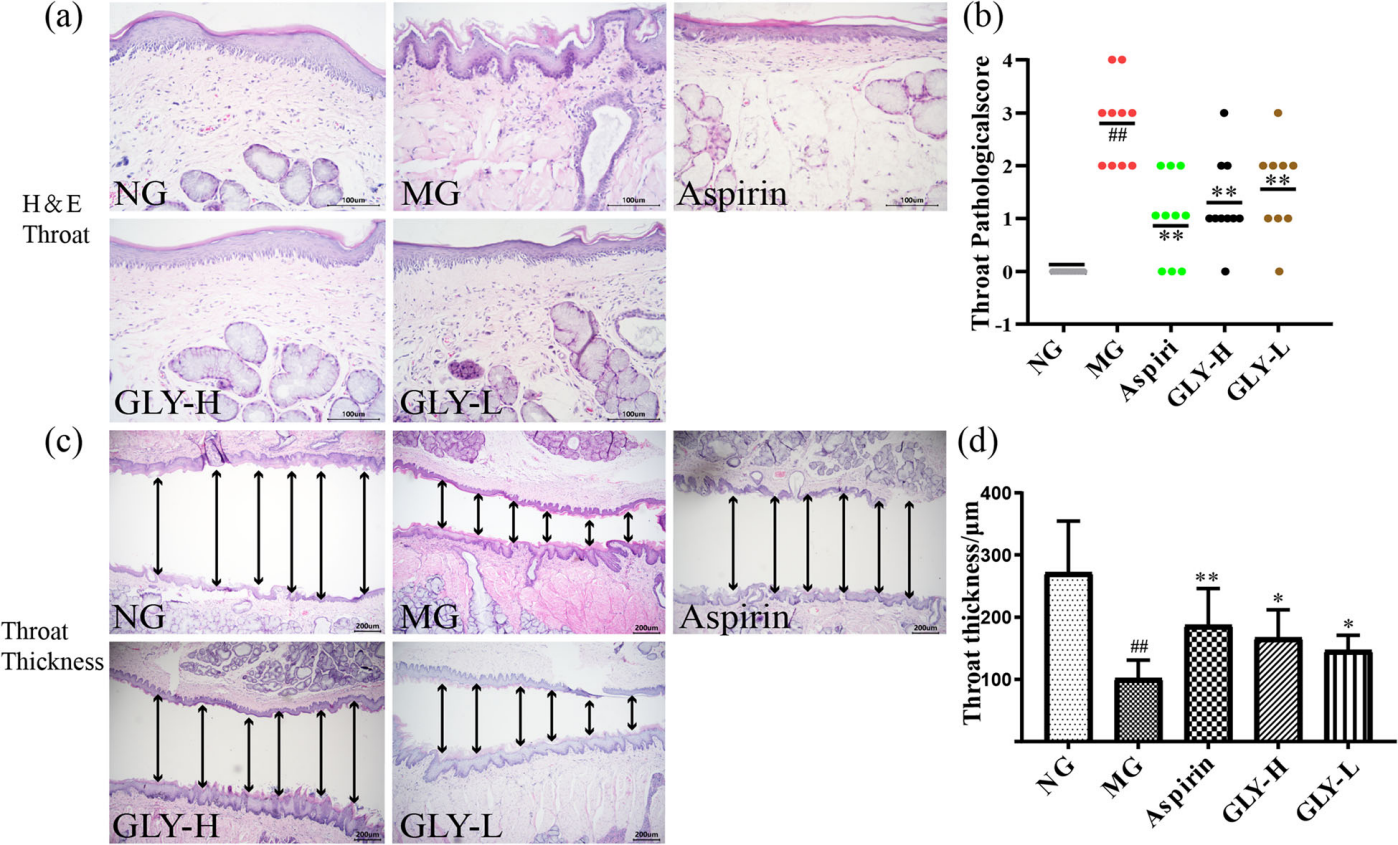
Effects of GLY on the throat pathology in CP rats. (**a**, **b**) Throat pathology by H&E staining (×400) and pathological score. (**c**, **d**) Throat thickness by H&E staining (×100) and analysis. ^#^*p *< 0.05, ^##^*p* < 0.01 compared with normal group; *p < 0.05, **p < 0.01 compared with model group, n=10

Compared the normal group, the ammonia-induced model group exhibited significant inflammatory cell infiltration as well as shedding of compound squamous epithelium, and there were significant subepithelial studs (Fig. 4a). However, in good agreement with our prior findings, the GLY-treated groups had less inflammation and less congestion of the pharynx (Fig. 4a). Indeed, GLY-H and GLY-L dramatically alleviated the pathological score (*P* < 0.01) (Fig. 4b). Further, compared with the normal group, under megascopic field of view in the throat of H&E staining, the throat mucosal space and throat thickness in the model group was significantly reduced (*P* < 0.01) (Fig. 4c and d), and GLY-H and GLY-L significantly increased the throat mucosal space and throat thickness (*P* < 0.05) (Fig. 4c and d), compared with model group. These data suggested that GLY helps reverse throat pathology in the ammonia-induced CP model in rats.

### GLY decreased the serum cytokine contents of IL-1β, IL-6, TNF- α, and PGE2 in ammonia-induced CP rats

**Fig. 5:**
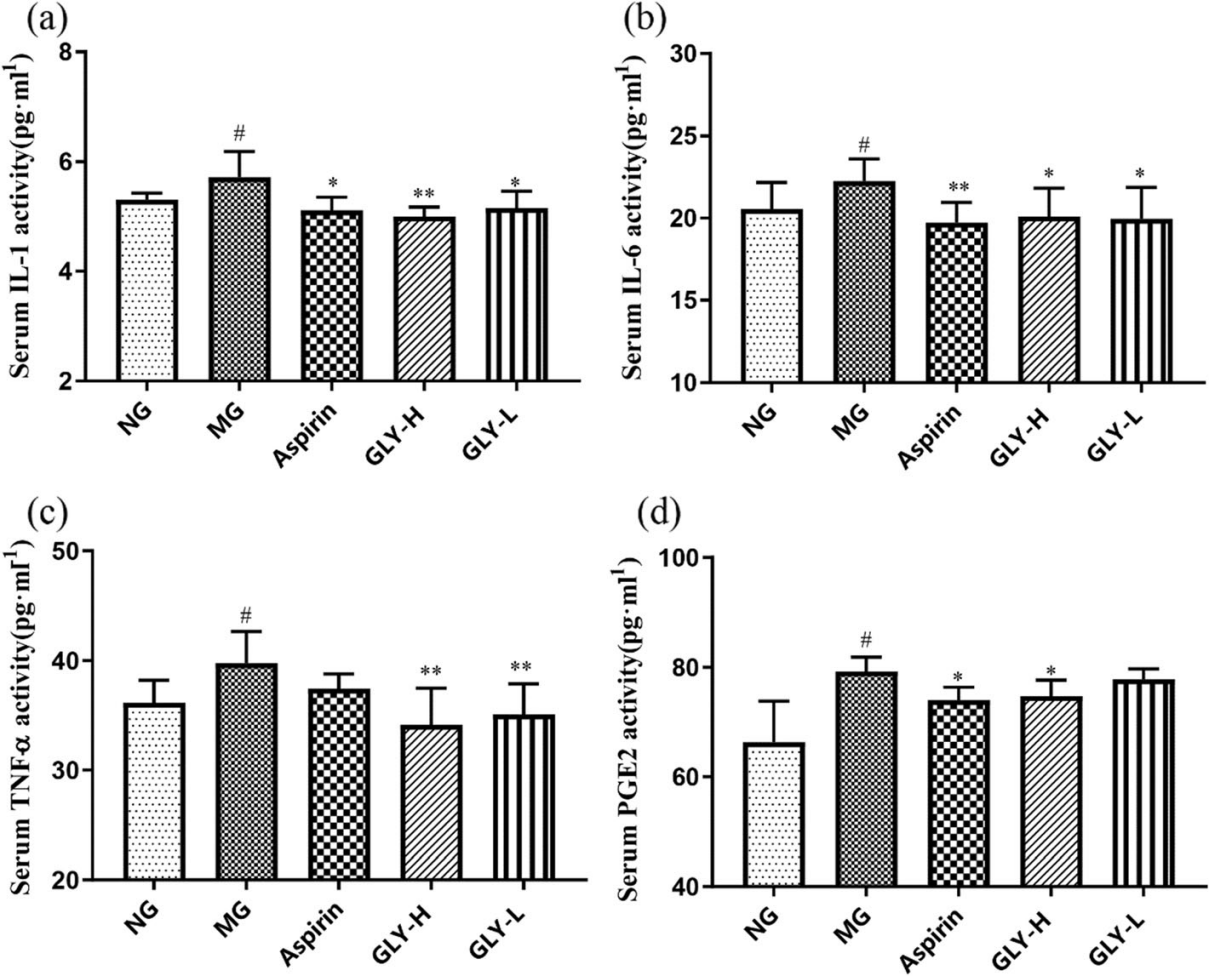
Effects of GLY on the serum inflammatory cytokines in CP rats. (**a**) The serum levels of IL-1β. (**b**) The serum levels of IL-6. (**c**) The serum levels of TNF-α. (**d**) The serum levels of PEG2. ^#^*p* < 0.05, ^##^*p* < 0.01 compared with normal group; *p < 0.05, **p < 0.01 compared with model group, n = 10

In the model group, the concentrations of serum inflammatory factors IL-1β, IL-6, PGE2, and TNF-α were obviously increased compared with the normal group (*P* < 0.05). However, GLY-H and GLY-L significantly reduced the concentration of IL-1β, IL-6, and TNF-α (*P* < 0.05, 0.01) (Fig. 5a-c), compared with the model control group. Further, GLY-H significantly reduced the levels of PGE2 (*P* < 0.05) (Fig. 5d), compared with the model control group. These data indicated that GLY acts through anti-inflammatory properties, leading to a reduction in serum inflammatory cytokines in rats with ammonia-induced CP.

### GLY decreased inflammatory signaling in the throat of rats with ammonia-induced CP

**Fig. 6:**
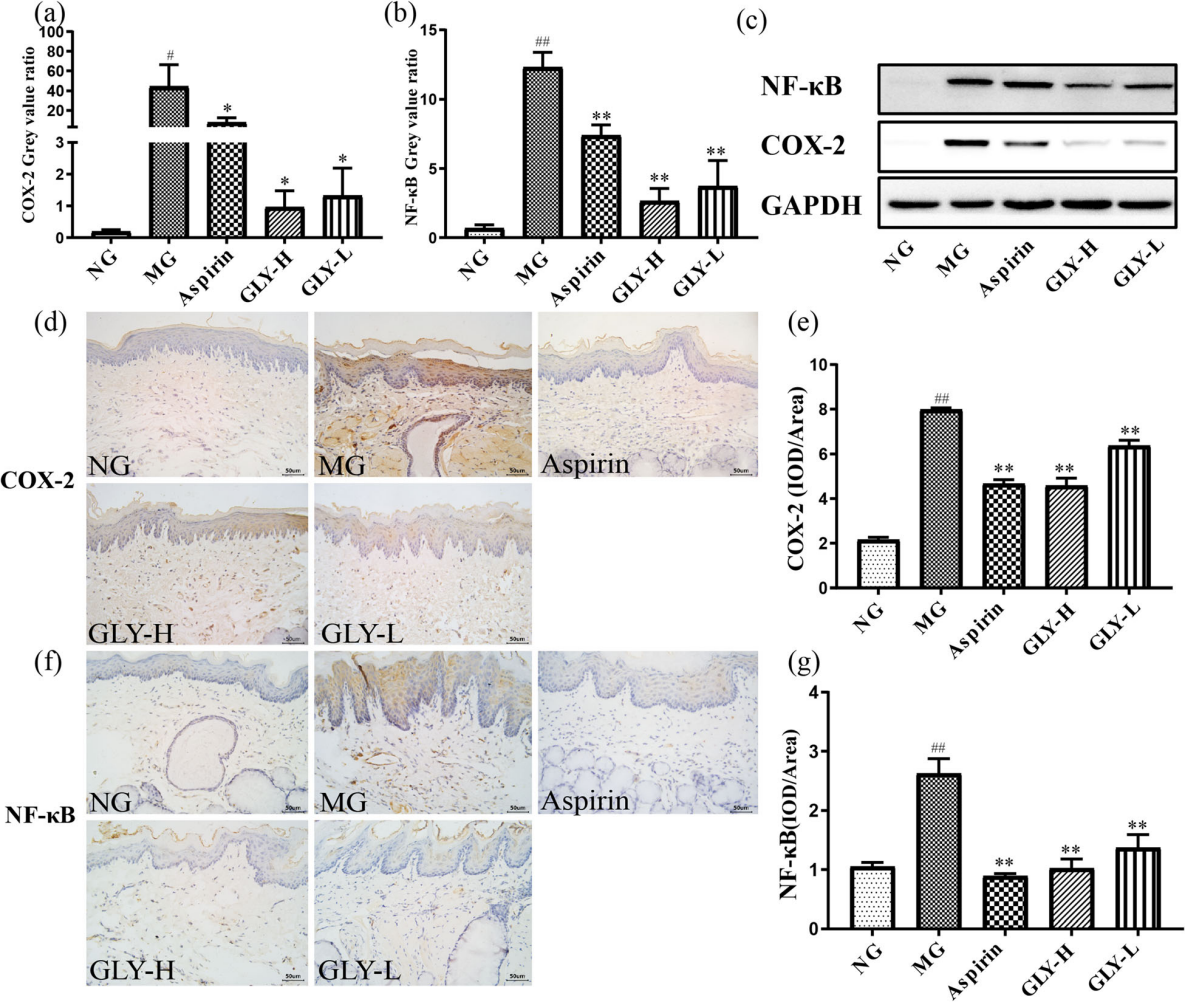
Effects of GLY on the COX-2 and NF-κB p65 expression of the throat in CP rats. (**a**, **b**) COX-2 and NF-κB p65 expression in the throat by the Western blot analysis. (**c**) Representative photomicrograph of COX-2 and NF-κB p65 protein expression by Western blot. (**d**) Representative photomicrograph of COX-2 protein expression by immunohistochemistry (IHC) in the throat (× 400). (**e**) Semiquantitative analysis of the of COX-2 protein expression by immunohistochemistry (IHC). (**f**) Representative photomicrograph of NF-κB p65 protein expression by immunohistochemistry (IHC) in the throat (× 400). (**g**) Semiquantitative analysis of the of NF-κB p65 protein expression by immunohistochemistry (IHC). ^#^*p* < 0.05, ^##^*p* < 0.01 compared with normal group; *p < 0.05, **p < 0.01 compared with model group, n = 10

As expected, Western blot studies found that the expression of COX-2 and NF-κB p65 in the throat of the model group markedly increased compared with the normal group (*P* < 0.05, 0.01). Compared with the model group, however, GLY-H and GLY-L significantly reduced expression levels of COX-2 and NF-κB p65 protein in the rat throat tissue (*P* < 0.05, 0.01) (Fig. 6a-c). Validating this finding, we found similar results after measuring the expression and localization of COX-2 and NF-κB p65 by immunohistochemistry, which is consistent with the Western blot results (*P* < 0.01) (Fig. 6d-g).

## Discussion

Pharyngitis (CP) is a highly prevalent inflammatory disease of the upper respiratory tract that poses significant decreases in quality of life. Although there are many antibiotics, glucocorticoids, and NSAIDs that can inhibit the exacerbation of pharyngitis, the side effects of these drugs can be significant to the point of intolerability over extended periods of use. At present, the treatment of CP with TCM is a popular and acceptable curative treatment [13, 31, 32]. TCM has relatively limited side-effects, does not adversely impact the microbiome, is inexpensive, and is thus a good candidate for long-term treatment, in comparison with antibiotics. GLY is a classical prescription of TCM that has been widely used in the clinic. In ancient China, GLY was reportedly used to treat various conditions [33, 34]. Recent research has investigated the potential benefits of using GLY as an adjuvant therapy in patients with throat disease by utilizing large-scale clinical databases and laboratory trials (*n* = 2542) [35]. It has been shown that patients with CP who are treated with GLY have a lower mortality and higher survival rate. The clinical efficacy of GLY is often noticeable after treatment, resulting in a gradual improvement in sore throat. The clinical bioavailability is more than 90% [15, 17, 36]. Surprisingly, the use of GLY is not typically associated with adverse reactions or side effects. Thus, TCM offers some potentially significant clinical benefits as complementary medicine [37]. However, although this formula has been used for the effective treatment of CP [15], its mechanism of action remains obscure. In this work, we evaluated the anti-inflammatory effects of GLY in two acute inflammation models, and evaluated the therapeutic effect of GLY in an ammonia-induced model of CP in rats.

Xylene-induced mice ear edema and carrageenan-induced rat paw edema are currently the two most widely used models of inflammation and are an effective tool for the study of novel anti-inflammatory drugs [24, 38, 39]. Several of the herbs included in GLY, such as HQ, SH and MD can significantly inhibit ear and foot swelling in mice [40–42]. Our current pharmacological results indicated that GLY had a significant anti-inflammatory effect at 2–6 h in the carrageenan-induced paw edema model in rats. In mice, the treated group had significantly reduced ear swelling and protection against the increase in ear thickness and blood circulation relative to the xylene-treated controls. Therefore, these findings suggest GLY has an anti-inflammatory effect.

Ammonia has been widely used to generate animal models of CP [10]. Using ammonia solution as a stimulant increases mucus secretion as well as counts of WBC, NEUT, LYMPH and MONO, pharyngeal mucosal congestion, and swelling. These symptoms are similar to the clinical manifestations of CP. SH, one of the components of GLY, possesses anti-pyretic, anti-inflammatory, and analgesic effects, and has been used to treat throat illnesses [43]. It has been reported that catalpol in DH and SDH have good anti-inflammatory effects and reduce the expression of NF-κB p65 [44]. PPY decreases the level of TNF-α and the expression of NF-κB and COX-2 [45, 46]. In good agreement with these prior studies, our results showed that GLY significantly decreased throat capillary dilation and secretion of mucus. GLY appeared to have a benefit by reducing serum inflammatory cytokines and decreasing the expression of COX-2 and NF-κB p65 proteins. These consequences confirm that GLY may have therapeutic effects for CP as a result of its anti-inflammatory effects.

Inflammation is associated with the initiation and development of pharyngitis. Modern pharmacology has extensively demonstrated that the production and release of PGE2 can exacerbate of inflammation. COX-2 is the main cyclooxygenase subtype. The expression of COX-2 promotes PGE2 production, which can not only induce inflammation, but also aggravate inflammation [47]. Selective therapeutics (such as NSAIDs) can mitigate inflammation by inhibiting PG [48]. Our experimental results confirmed this principle by finding that the expression of COX-2 protein in the throat tissue of the GLY-treated groups was decreased and the contents of PGE2 in the serum of rats decreased, relative to the model group. The pathological results suggested that the model group had a more severe inflammatory response in which there was shedding of the pharyngeal mucosa, diffuse inflammation, and the development of subepithelial studs. These observations indicate that the activation of COX-2 and subsequent PGE2 production and release promoted the development of pharyngitis. However, treatment with GLY inhibited this process.

The transcription factor NF-κB mediates inflammation by promoting the expression of specific genes related to promoting inflammation [49, 50]. NF-κB signal transduction is through two different pathways: the canonical pathway and non-canonical pathway. The canonical pathway promotes inflammatory reactions, and the non-canonical pathway promotes the maturation and differentiation of immune cells and secondary lymphoid organogenesis [51]. COX-2 is the main target of NF-κB. Multiple inflammatory stimuli and mediators increase the expression of COX-2 through the activation of NF-κB, thereby causing inflammation [52]. Studies have shown that TNF-α, IL-1β, and IL-6 are involved and overexpressed during the occurrence and development of chronic inflammatory diseases [53]. This is consistent with the results of chronic pharyngitis seen in ammonia-induced models in rats [54]. At the same time, IL-1β acts as an extracellular stimulating factor and can activate NF-κB to further amplify the inflammatory response [52]. Our studies found that the administration of GLY remarkably decreased the serum levels of TNF-α, IL-1β, and IL-6 and decreased the expression NF-κB level, which supports the anti-CP activity of GLY and suggested that this effect is at least partially mediated via the down-regulation of proinflammatory cytokines and NF-κB in CP rats.

The primary active compounds were identified from GLY and included naringin, neohesperidin, baicalin, and wogonoside. Naringin acts against the increased expression of NF-κB and inhibits the production and expression of inflammatory genes and inflammasomes [55, 56]. Shi has previously reported that paraquat leads to remarkably increased expression of NF-κB and COX-2, however these effects were decreased after treatment with neohesperidin [57]. Baicalin and wogonoside have strong antioxidant and anti-inflammatory effects through decreasing the expression of NF-κB and COX-2 [58–60]. Therefore, the effects of GLY in the context of CP are likely related to the combined actions of these components, but is a topic for further study.

## Conclusion

Our results revealed that GLY had significant anti-inflammatory effects in animal models of inflammation and CP, leading to improvements in pharyngeal inflammation. These effects appeared to be regulated through the NF-κB/COX-2 signaling pathway and through protection against increased expression of inflammatory cytokines induced by ammonia. These findings support the further evaluation of GLY as a potential drug for the treatment of CP and warrant further studies of its mechanisms of action. GLY may have significant clinical potential as an anti-inflammatory therapeutic for the treatment of inflammatory conditions such as pharyngitis.

## Supplementary information

**Additional file 1.**

## Data Availability

The datasets used and/or analyzed during the current study are available from the corresponding author on reasonable request.

## Abbreviations

CP: Chronic pharyngitis
GLY: Ganluyin
NSAIDs: Nonsteroidal anti-inflammatory drugs
TCM: Traditional Chinese Medicine
IL-1β: Interleukin 1β
IL-6: Interleukin 6
TNF-α: Tumor necrosis factor α
PGE2: Prostaglandin E2
COX-2: Cyclooxygenase-2
NF-κB p65: Nuclear factor kappa-B p65
ICR: Institute of Cancer Research
SD: Sprague Dawley
ELISA: Enzyme-linked immunesorbent assay
HPLC: High performance liquid chromatography
DOP: Dendrobium officinale polysaccharide
NLRP3: Nucleotide binding oligomerization domain-like receptor protein 3
UC: Ulcerative Colitis
MAP K14: Mitogen-activated protein kinase 14
EGFR: Epidermal growth factor receptor.
SELE: E-Selectin.
IOD: Integrated option density.
WBC: White blood cell.
NEUT: Neutrophil.
LYMPH: Lymphocyte.
MONO: Monocyte.
EDTA: Ethylene Diamine Tetraacetic Acid.
H&E: Hematoxylin and eosin.
RIPA: Radio-Immunoprecipitation Assay.
AKT: Protein kinase B.
ERK: Extracellular regulated protein kinases.
GAPDH: Glyceraldehyde-3-phosphate dehydrogenase.
MeOH: Methyl alcohol.
