# Supplementary material for: Anti-inflammatory effect of Ganluyin, a Chinese classic prescription, in chronic pharyngitis rat model
Source: BMC Complement Med Ther. 2020 Aug 28;20:265. doi: 10.1186/s12906-020-03057-5 (PMC7456022; doi:10.1186/s12906-020-03057-5)
Supplement: Supplementary file 1 — Additional file 1. [file 12906_2020_3057_MOESM1_ESM.docx]

**Supplementary Material**

**Antiinflammatory effect of Ganluyin, a Chinese classic prescription, in chronic pharyngitis rat model**

Ye-Hui Chen ^1†^, Rong Luo ^1†^, Shan-Shan Lei^1^, Bing Li^1^, Fu-Chen Zhou^1^, Hui-Ying Wang^1^, Xue Chen^1^, Xinglishang He^1^, Yu-Zhi Wang^1^, Liang-Hui Zhan^1^, Ting-Ting Lu^1^, Jie Su^2^, Qiao-Xian Yu^3^, Bo Li^1*^, Gui-Yuan Lv^2*^, Su-Hong Chen^1*^

*^1^ Zhejiang University of Technology, Hangzhou, Zhejiang 310014, PR China;*

*^2^ Zhejiang Chinese Medical University, Hangzhou, Zhejiang 310053, PR China*

*^3^ Zhejiang Senyu Co., Ltd, Yiwu, Zhejiang, 322099, PR China*

^1†^ These authors contributed equally to this work.

∗Corresponding authors:

E-mail addresses: boli19861023@163.com (B. Li), zjtcmlgy@163.com (G. Y. Lv), chensuhong@zjut.edu.cn (S. H. Chen).

**Contents:**

**Table S1.** The composition and full details of GLY

**Table S1.** The composition and full details of GLY

| No. | Chinese name | Abbreviation | Botanical name | Plant family | Part used | Original proportions | Weight (g) | Batch numbers |
| --- | --- | --- | --- | --- | --- | --- | --- | --- |
| 1 | Huang Qin | HQ | *Scutellaria Baicalensis* | Labiatae | Root | 1 | 7.4 | 180304 |
| 2 | Shi Hu | SH | *Dendrobii Caulis* | Orchidaceae | Stem | 1 | 7.4 | 181126 |
| 3 | Di Huang | DH | *Rehmannia Glutinosa* | Scrophulariaceae | Root | 1 | 7.4 | 1901019 |
| 4 | Shu Di Huang | SDH | *Radix Rehmanniae* | Scrophulariaceae | Root | 1 | 7.4 | 1812016 |
| 5 | Tian Dong | TD | *Asparagus Cochinchinensis* | Liliaceae | Root | 1 | 7.4 | 1901016 |
| 6 | Mai Dong | MD | *Ophiopogon Japonicus* | Liliaceae | Root | 1 | 7.4 | 1812059 |
| 7 | Yin Chen | YC | *Artemisia Capillaris* | Compositae | Aboveground | 1 | 7.4 | 1808024 |
| 8 | Pi Pa Ye | PPY | *Eriobotrya Japonica* | Rosaceae | Leaf | 1 | 7.4 | 180428 |
| 9 | Zhi Qiao | ZQ | *Fructus Aurantii* | Rutaceae | Fruit | 1 | 7.4 | 1810052 |
| 10 | Zhi Gan Cao | ZGC | *Glycyrrhiza Uralensis* | Leguminosae | Root | 1 | 7.4 | 1902013 |

**Fig. S1** Identification of main peak


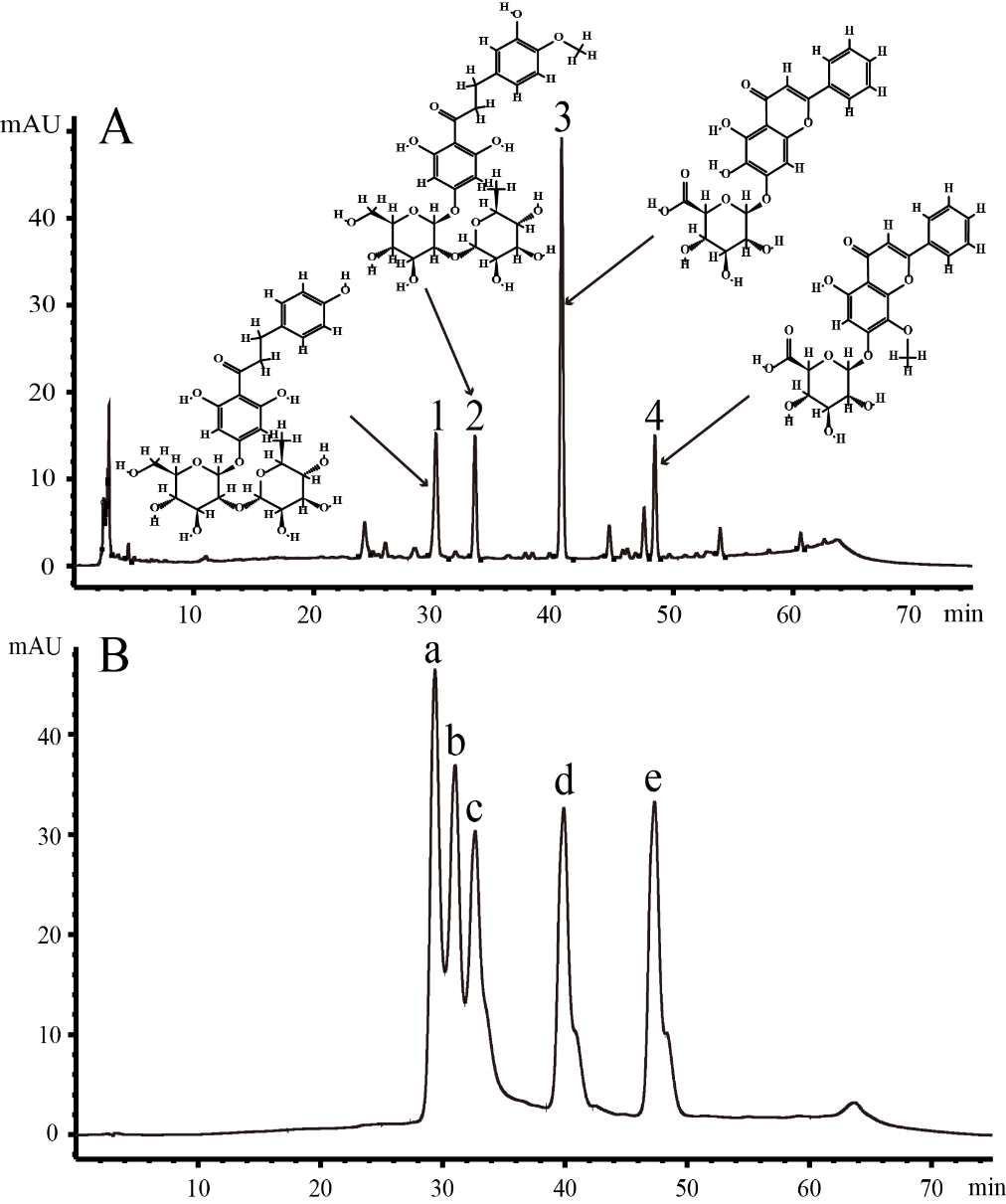


**Fig. S1**. GLY water extract. 1 = naringin, 2 = neohesperidin, 3 = baicalin, 4 = wogonoside. (A) Comparative with mixed reference standards solution. a = naringin standard, b = hesperidin standard, c = neohesperidin standard, d = baicalin standard, e = wogonoside standard. (B)


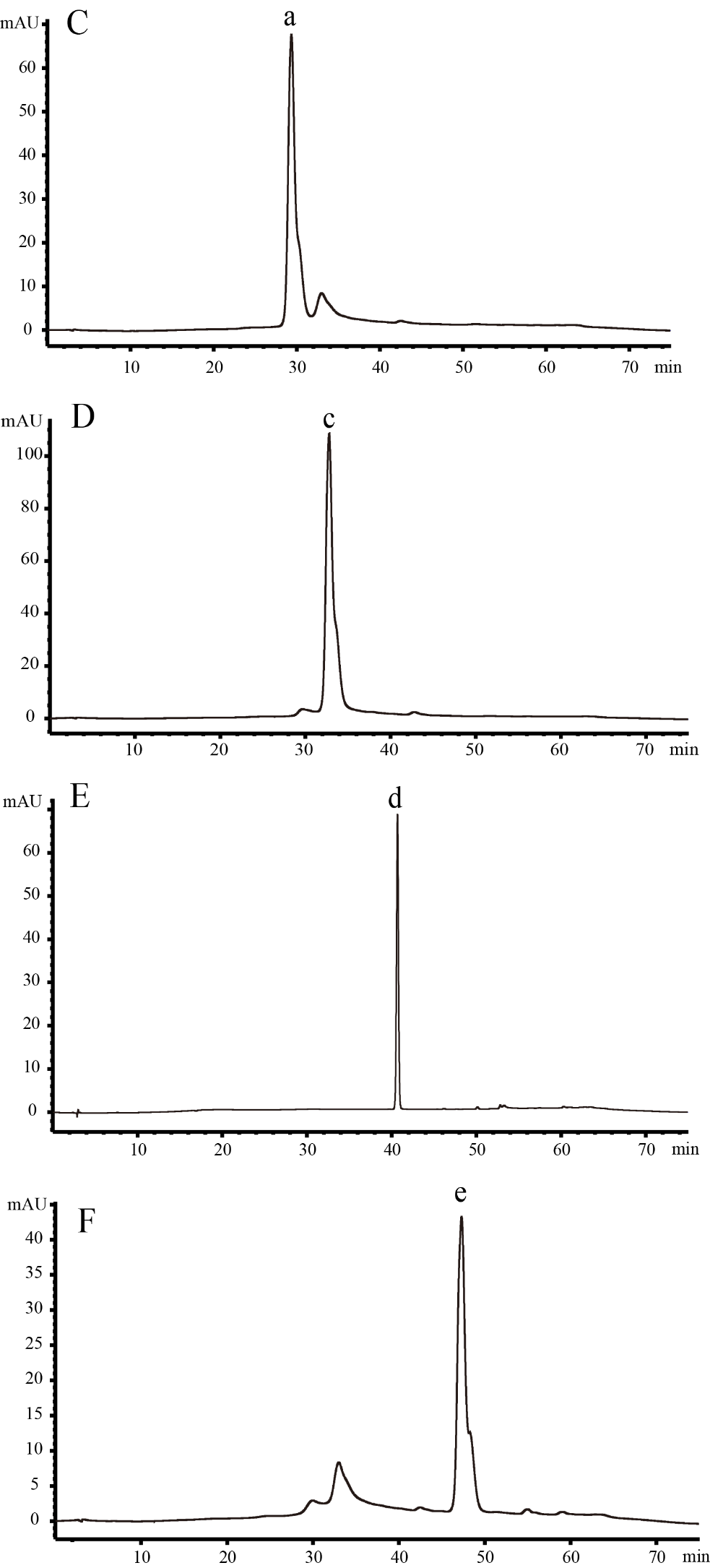


**Fig. S2**. Comparative with single reference standards solution. a = naringin standard, (C) c = neohesperidin standard, (D) d = baicalin standard, (E) e = wogonoside standard. (F)
